# Structural Dynamics and Susceptibility of Aminonaphthoquinone–Chalcone Hybrids Against Human Tyrosinase

**DOI:** 10.3390/ijms27167328

**Published:** 2026-08-17

**Authors:** Sahachai Sabuakham, Napat Nuramrum, Thanyada Rungrotmongkol, Ratchanok Pingaew, Panupong Mahalapbutr

**Affiliations:** 1 Department of Biochemistry, Center for Translational Medicine, Faculty of Medicine, Khon Kaen University, Khon Kaen 40002, Thailand; 2 Program in Bioinformatics and Computational Biology, College of Interdisciplinary and Integrative Studies, Chulalongkorn University, Bangkok 10330, Thailand; 3 Center of Excellence in Structural and Computational Biology, Department of Biochemistry, Faculty of Science, Chulalongkorn University, Bangkok 10330, Thailand; 4 Department of Chemistry, Faculty of Science, Srinakharinwirot University, Bangkok 10110, Thailand

**Keywords:** tyrosinase, aminonaphthoquinone–chalcone hybrids, molecular docking, molecular dynamics simulation, molecular modeling

## Abstract

Tyrosinase is a key enzyme that catalyzes the rate-limiting step in melanogenesis. Although numerous mushroom-derived tyrosinase inhibitors have been identified, many exhibit limited efficacy against human tyrosinase. In this study, *in silico* approaches were employed to evaluate the inhibitory potential of aminonaphthoquinone–chalcone hybrids against human tyrosinase. Molecular docking analysis revealed that all 10 hybrids showed higher binding affinity than kojic acid. Among them, six hybrids (**3**, **4**, **5**, **7**, **9**, and **10**) displayed higher binding affinity than the others and interacted with amino acid residues within the active site of tyrosinase. Molecular dynamics simulations revealed that the six selected hybrids maintained stable binding to human tyrosinase throughout the simulations. Among them, compound **9** exhibited the highest structural stability, consistent with its most favorable MM/PBSA-predicted binding free energy and the greatest number of key residue interactions (K306, K334, S358, S360, N364, H367, I368, S375, Q376, and V377). Van der Waals interactions were identified as the predominant driving force governing ligand–protein binding in all complexes. These findings provide atomistic insights into the interactions between aminonaphthoquinone–chalcone hybrids and human tyrosinase and may facilitate the future development of novel human tyrosinase inhibitors.

## 1. Introduction

**Figure 1:**
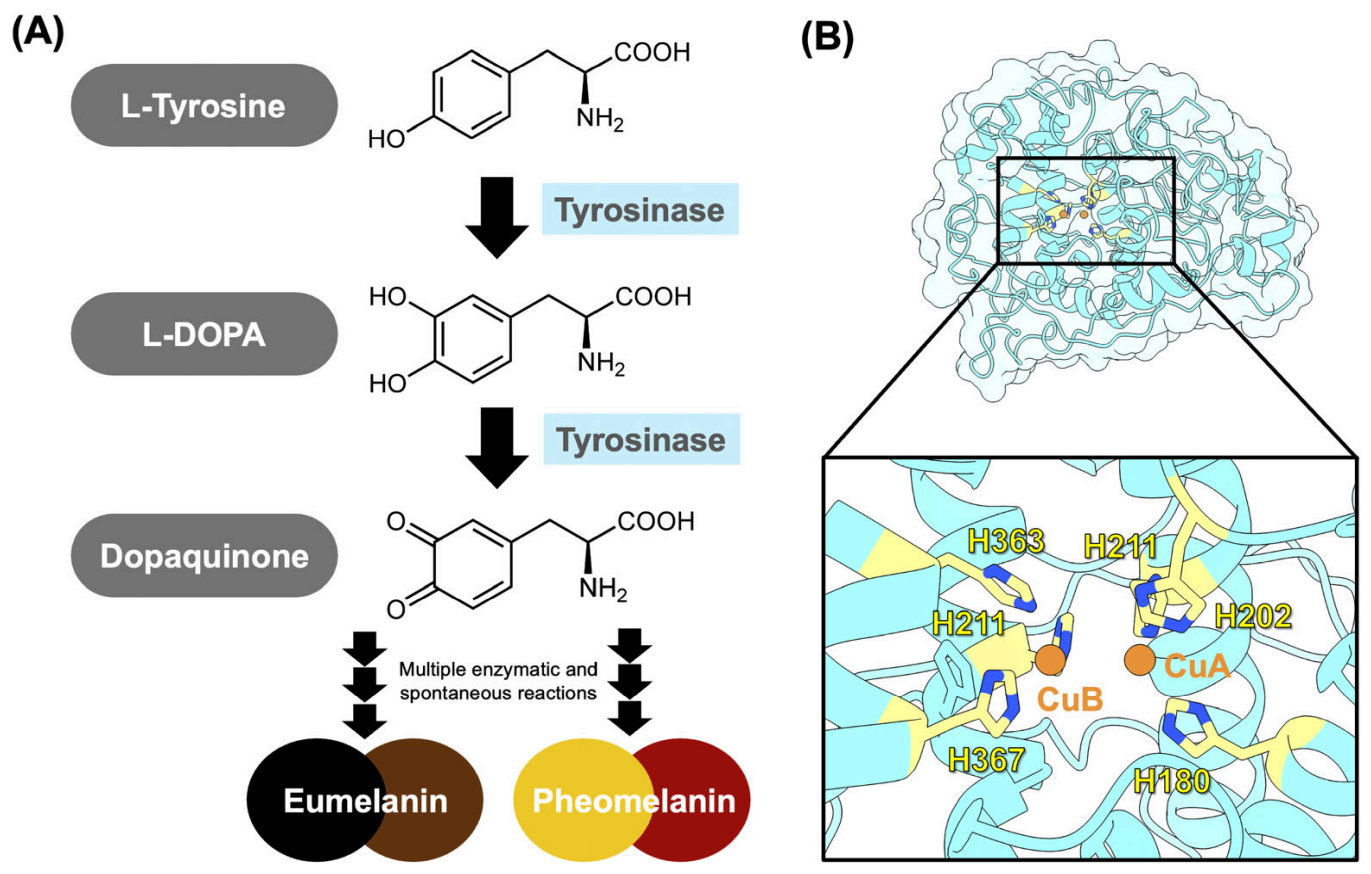
(**A**) Melanogenesis pathway [13]. (**B**) Homology model of human tyrosinase. The catalytic histidine residues and copper atoms are shown in yellow and orange, respectively.

Tyrosinase, the rate-limiting enzyme in melanogenesis, catalyzes the conversion of L-tyrosine to L-DOPA and subsequently to dopaquinone, which is further processed through several enzymatic and spontaneous reactions to produce melanin (Figure 1A) [1,2]. In addition, tyrosinase-related protein 1 (TRP1) catalyzes the oxidation of 5,6-dihydroxyindole-2-carboxylic acid (DHICA), which is generated from dopachrome by tyrosinase-related protein 2 (TRP2), thereby contributing to eumelanin synthesis [3,4]. The active site of tyrosinase contains a coupled binuclear copper center (Figure 1B) that binds molecular oxygen to form an oxy-tyrosinase intermediate, which initiates the monooxygenation reaction [5,6]. In contrast, TRP1 and TRP2 require zinc ions for melanin biosynthesis [4,7,8].

Excessive melanin production and accumulation contribute to various hyperpigmentation disorders, including solar lentigines, melasma, freckles, and post-inflammatory hyperpigmentation [2,9]. Therefore, tyrosinase has emerged as a primary target for the development of depigmenting agents [10]. Although several tyrosinase inhibitors have been developed, many are associated with significant adverse effects [2]. For example, hydroquinone and arbutin may cause skin irritation and contact dermatitis [11,12], whereas kojic acid is chemically unstable, particularly during storage [13]. Furthermore, the development of most tyrosinase inhibitors has relied on mushroom tyrosinase, limiting their efficacy and translational potential against human tyrosinase because of structural differences between the two enzymes [14]. Therefore, there remains a critical need to discover novel, potent inhibitors that specifically target human tyrosinase.

Recent studies have identified several promising inhibitors of human tyrosinase. Mann et al. screened a library of 50,000 compounds and compared the active hits with established depigmenting agents. Among the tested compounds, Thiamidol™ (isobutylamido thiazolyl resorcinol) emerged as the most potent human tyrosinase inhibitor, exhibiting an IC_50_ value of 1.1 μmol/L [15]. Hassan et al. identified vanillin and coumarin derivatives (V7 and C9) as potent human tyrosinase inhibitors, showing higher binding affinity than kojic acid and arbutin [16]. Furthermore, anthocyanin-rich extracts from the seed coats of black soybean were shown to inhibit human tyrosinase activity [17].

Several naphthoquinone- and chalcone-based compounds have been reported to possess anti-tyrosinase activity. Among naphthoquinone derivatives, plumbagin and lawsone inhibited tyrosinase activity in B16F10 mouse melanoma cells [18,19]. β-Lapachone not only suppressed tyrosinase activity but also reduced melanin synthesis by downregulating the expression of microphthalmia-associated transcription factor (MITF), tyrosinase, and tyrosinase-related proteins [20]. Chalcone derivatives have likewise demonstrated potent anti-tyrosinase activity. For example, 6-hydroxy-2-(3,4,5-trimethoxybenzylidene)-3,4-dihydronaphthalen-1(2H)-one (C2), inhibited tyrosinase with an IC_50_ value of 8.8 μM, slightly outperforming kojic acid (IC_50_ = 9.7 μM) [21]. In addition, chalcone–hydroxypyridinone hybrids (1a and 1d) potently inhibited both the monophenolase activity (IC_50_ = 3.07 μM and 2.25 μM, respectively) and diphenolase activity (IC_50_ = 17.05 μM and 11.70 μM, respectively) of tyrosinase [22]. Similarly, butein and homobutein exhibited inhibitory activity against tyrosinase, with IC_50_ values of 10.88 and 14.78 μM for monophenolase activity and 15.20 and 12.36 μM for diphenolase activity, respectively [23]. 

Based on these findings, the molecular hybridization of naphthoquinone and chalcone scaffolds represents a rational strategy for developing novel tyrosinase inhibitors by integrating the complementary pharmacological features of both scaffolds into a single molecular framework. In particular, aminonaphthoquinone–chalcone hybrids incorporate structural features associated with potent tyrosinase inhibition, including extended aromatic π-systems, multiple hydrogen-bond donor and acceptor functionalities [24,25], and trimethoxy substitution patterns [21]. These characteristics are consistent with the structural requirements for effective tyrosinase inhibition reported in previous studies. Nevertheless, despite the well-documented anti-tyrosinase activities of naphthoquinone and chalcone derivatives, the inhibitory potential of aminonaphthoquinone–chalcone hybrids against human tyrosinase has not yet been investigated.

**Figure 2:**
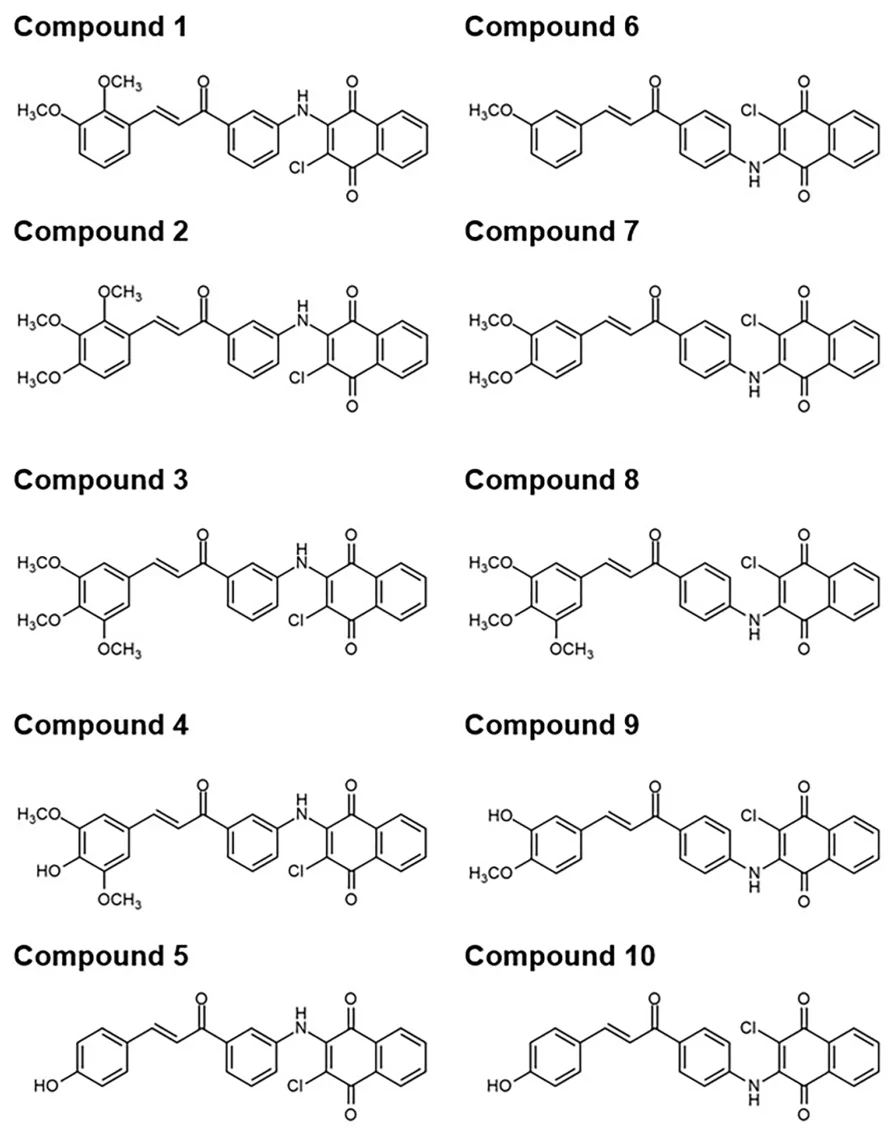
Chemical structures of the 10 aminonaphthoquinone–chalcone hybrids reported in a previous study [26].

In this study, molecular docking, molecular dynamics (MD) simulations, and molecular mechanics/Poisson–Boltzmann surface area (MM/PBSA) binding free energy (Δ*G*_bind_) calculations were employed to investigate the inhibitory potential of aminonaphthoquinone–chalcone hybrids (Figure 2) against human tyrosinase. The findings provide molecular insights into the binding mechanisms of these hybrids and may facilitate the rational design and development of novel human tyrosinase inhibitors based on the aminonaphthoquinone–chalcone scaffold.

## 2. Results and Discussion

### 2.1. Molecular Docking

To investigate the binding affinity and binding pattern of the studied compounds against human tyrosinase, molecular docking was performed using the CB-Dock2 web server. Kojic acid, a well-established tyrosinase inhibitor, was used as a positive control and exhibited a predicted binding energy of −5.0 kcal/mol (Supplementary Figure S1). All 10 aminonaphthoquinone–chalcone hybrids demonstrated higher predicted binding affinity than kojic acid. As shown in Table 1, the binding energy of the hybrids in complex with human tyrosinase was in a range of −9.2 to −7.5 kcal/mol. Among them, compound **4** showed the lowest binding energy (−9.2 kcal/mol), followed by compound **10** (−8.5 kcal/mol) and compounds **3**, **5**, **7**, and **9** (−8.4 kcal/mol), all of which exhibited lower binding energies than the remaining derivatives. These results suggest that compounds **3**, **4**, **5**, **7**, **9**, and **10** possess higher predicted binding affinity toward human tyrosinase than the other hybrids.

**Figure 3:**
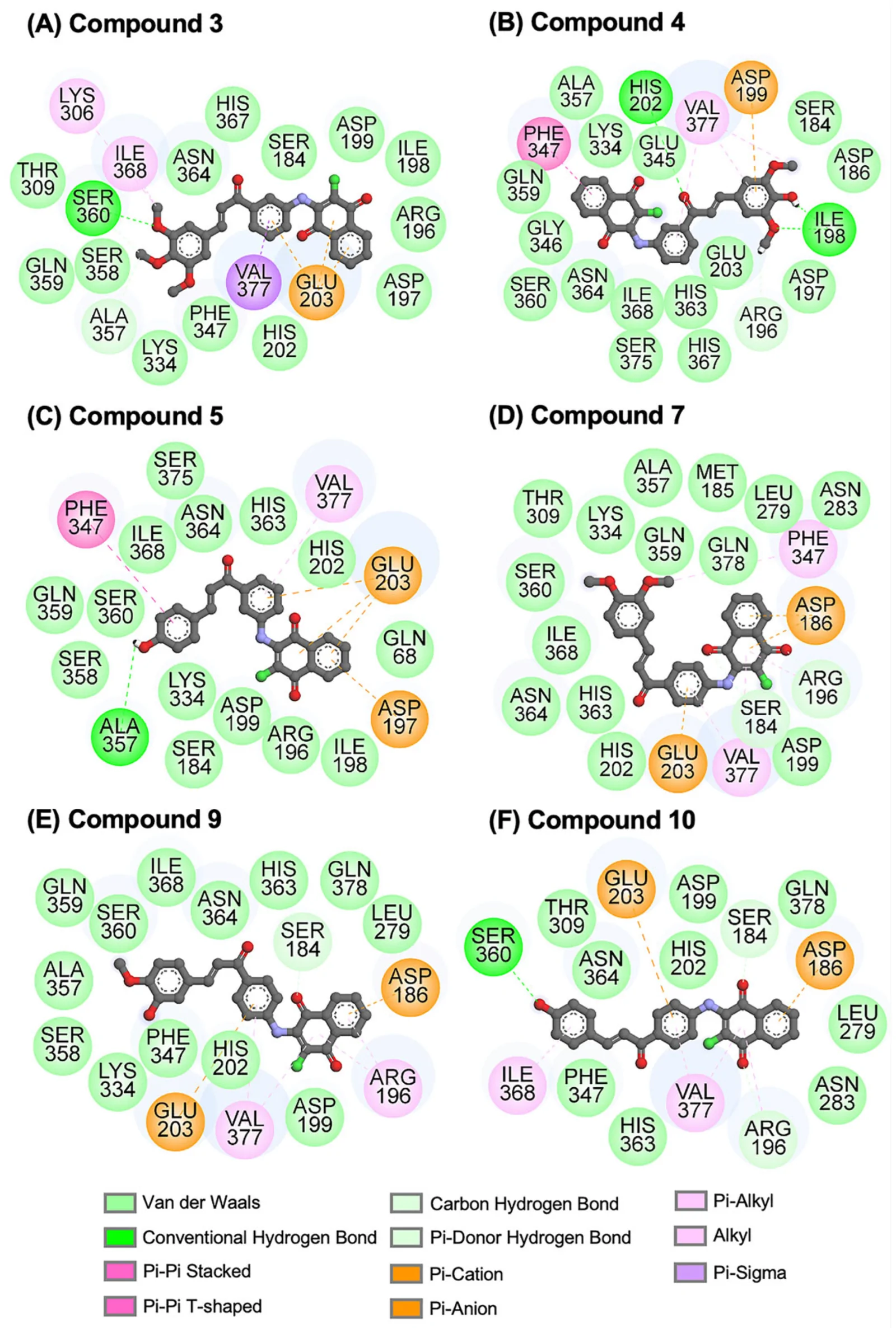
2D interaction profile of six aminonaphthoquinone–chalcone hybrids in complex with human tyrosinase predicted by molecular docking.

The two-dimensional (2D) interactions between the ligands and human tyrosinase were further investigated. As displayed in Figure 3A, the methoxy group of compound **3** formed a hydrogen bond (H-bond) with residue S360. Compound **4** (Figure 3B) formed H-bonds with residues I198 and H202, while its aromatic rings established Pi interactions with residues D199, F347, and V377. The hydroxyl (−OH) group of compound **5** formed an H-bond with residue A357 (Fig-ure 3C). Similarly, the −OH group of compound **10** (Figure 3F) formed an H-bond with S360, while its aromatic rings established Pi interactions with residues D186, E203, I368, and V377.

Although no H-bonds were detected for compounds **7** and **9** in complex with human tyrosinase (Figure 3D,E), both compounds established Pi interactions with several residues. Compound **7** interacted with D186, E203, F347, and V377, whereas compound **9** interacted with D186, R196, E203, and V377. Notably, residue V377 was identified as a key binding residue in all ligand–human tyrosinase complexes. This observation is consistent with previous studies showing that V377 is one of the key amino acid residues involved in the binding of thujaplicins and Thiamidol™ to human tyrosinase [27,28]. 

The presence of methoxy and −OH groups in the aromatic moieties of the potent compounds (**3**, **4**, **5**, **7**, **9**, and **10**) potentially formed hydrogen bonds, van der Waals (vdW) forces, and Pi interactions with the human tyrosinase. These findings align well with previous reports stating that the presence of −OH groups enhanced binding affinity and increased their anti-tyrosinase activity [24,25].

It should be noted that all six studied hybrids interacted with amino acid residues located within the active site of human tyrosinase, consistent with the previously reported binding of amphotericin B [29], kojic acid, arbutin [16], coumarin (compound C9) [16], and vanillin (compound V7) [16] to human tyrosinase. Based on these findings, the six aminonaphthoquinone–chalcone hybrids were selected for further MD simulations, identification of key binding residues, Δ*G*_bind_ calculations, and drug-likeness prediction.

### 2.2. Structural Stability and Compactness of Protein–Ligand Complex

**Figure 4:**
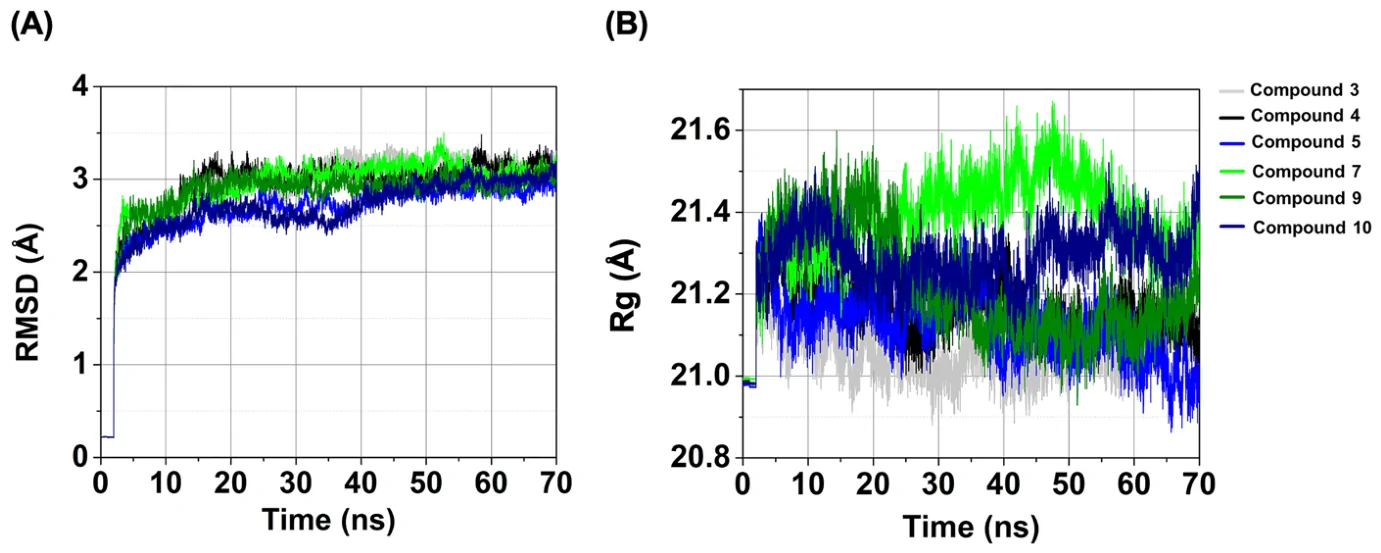
Time evolution of (**A**) RMSD and (**B**) Rg for six aminonaphthoquinone–chalcone hybrid/human tyrosinase complexes obtained from MD simulations.

The root-mean-square deviation (RMSD) was calculated to assess structural stability of the complexes throughout the simulation period [30]. As shown in Figure 4A, the RMSD values of all complexes gradually increased during the initial stage of the simulation and reached equilibrium after approximately 60 ns. Thereafter, all complexes maintained relatively stable fluctuations, with average RMSD values ranging from ~2.91 to 3.14 Å, as calculated over the final 10 ns of the simulation. The average RMSD values were 3.14 ± 0.06, 3.14 ± 0.07, 2.91 ± 0.06, and 3.07 ± 0.07 Å for compounds **3**, **4**, **5**, and **7**, respectively, in complex with human tyrosinase. Notably, the complex with compound **5**, followed by those with compounds **9** and **10**, exhibited lower RMSD values than the other systems, suggesting greater structural stability in the aqueous environment.

We further investigated the compactness of the ligand–protein complexes using the radius of gyration (Rg) as an indicator of structural compactness during complexation [31]. As shown in Figure 4B, the Rg values of all complexes remained relatively stable during the final 10 ns of the simulation, ranging from approximately 20.9 to 21.5 Å. Notably, the complexes of compounds **3**, **4**, **5**, and **9** with human tyrosinase exhibited lower average Rg values than the other complexes. In particular, the compound **5**–human tyrosinase complex showed the lowest average Rg value (21.02 ± 0.06 Å), suggesting the highest degree of structural compactness among the studied complexes.

These findings regarding the structural stability and compactness suggest that five compounds (**3**, **4**, **5**, **9**, and **10**), particularly compound **9**, formed more compact and structurally stable complexes with human tyrosinase than the other complexes. The corresponding rolling-average RMSD and Rg profiles are provided in the Supporting Information (Figures S2–S5). Overall, the MD simulation results demonstrated that compounds **4**, **7**, and particularly **9** maintained stable conformations and favorable interactions within the active site (Figure S6).

### 2.3. Atomic Contacts and H-Bonds

**Figure 5:**
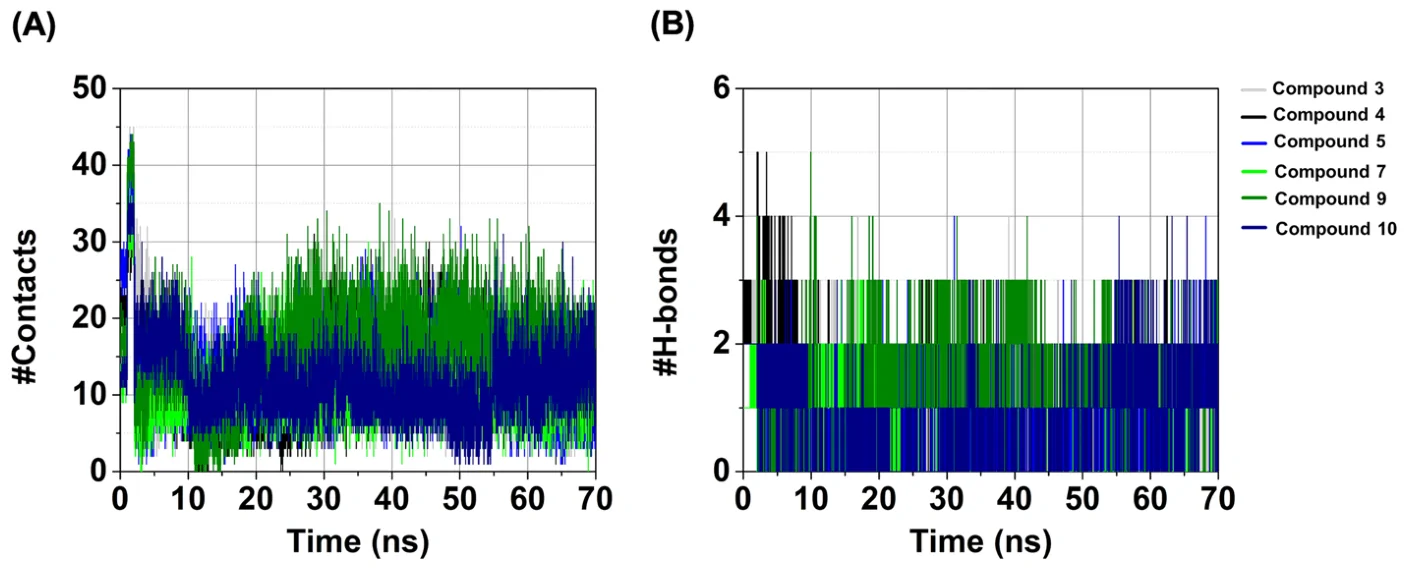
Time evolution of (**A**) #Contacts and (**B**) #H-bonds for six aminonaphthoquinone–chalcone hybrids in complex with human tyrosinase obtained from MD simulations.

Atomic contacts contribute to the binding affinity of protein–ligand complex [32]. The number of atomic contacts (#Contacts) between the ligands and human tyrosinase was calculated by counting the residues within 3.5 Å of each ligand. As depicted in Figure 5A, the average #Contacts values were 11 ± 4, 12 ± 4, 9 ± 4, 11 ± 4, 16 ± 4, and 13 ± 4 for compounds **3**, **4**, **5**, **7**, **9**, and **10** in complex with human tyrosinase, respectively. Among these complexes, compound **9**–human tyrosinase complex exhibited the highest #Contacts, followed by the complexes with compounds **10** and **4**.

H-bonds are essential non-covalent interactions that play a pivotal role in ligand–protein binding [33]. In this study, H-bonds were identified based on a donor–acceptor distance of ≤3.5 Å and a donor–hydrogen–acceptor (HBD–H···HBA) angle of ≥120° [34] during the last 10 ns of the simulation. The results are presented in Figure 5B. Among the investigated complexes, the compound **9**–human tyrosinase complex exhibited the highest average number of H-bonds (#H-bonds; 1.6 ± 0.6), followed by the compound **10** (1.4 ± 0.8) and compound **4** (1.0 ± 0.4) complexes. The rolling-average analyses of #Contacts and #H-bonds are provided in the Supporting Information (Figures S7–S10).

The H-bond occupancy analysis (Supplementary Table S1) revealed that compound **4** formed the most persistent H-bond with human tyrosinase (97.85% occupancy at residue S360), followed by compound **9** (86.70% occupancy at residue S375). Compound **10** also established two stable H-bonds with S375 (62.85% occupancy) and A357 (54.65% occupancy). In contrast, no H-bonds were detected for the compound **7**–human tyrosinase complex.

**Figure 6:**
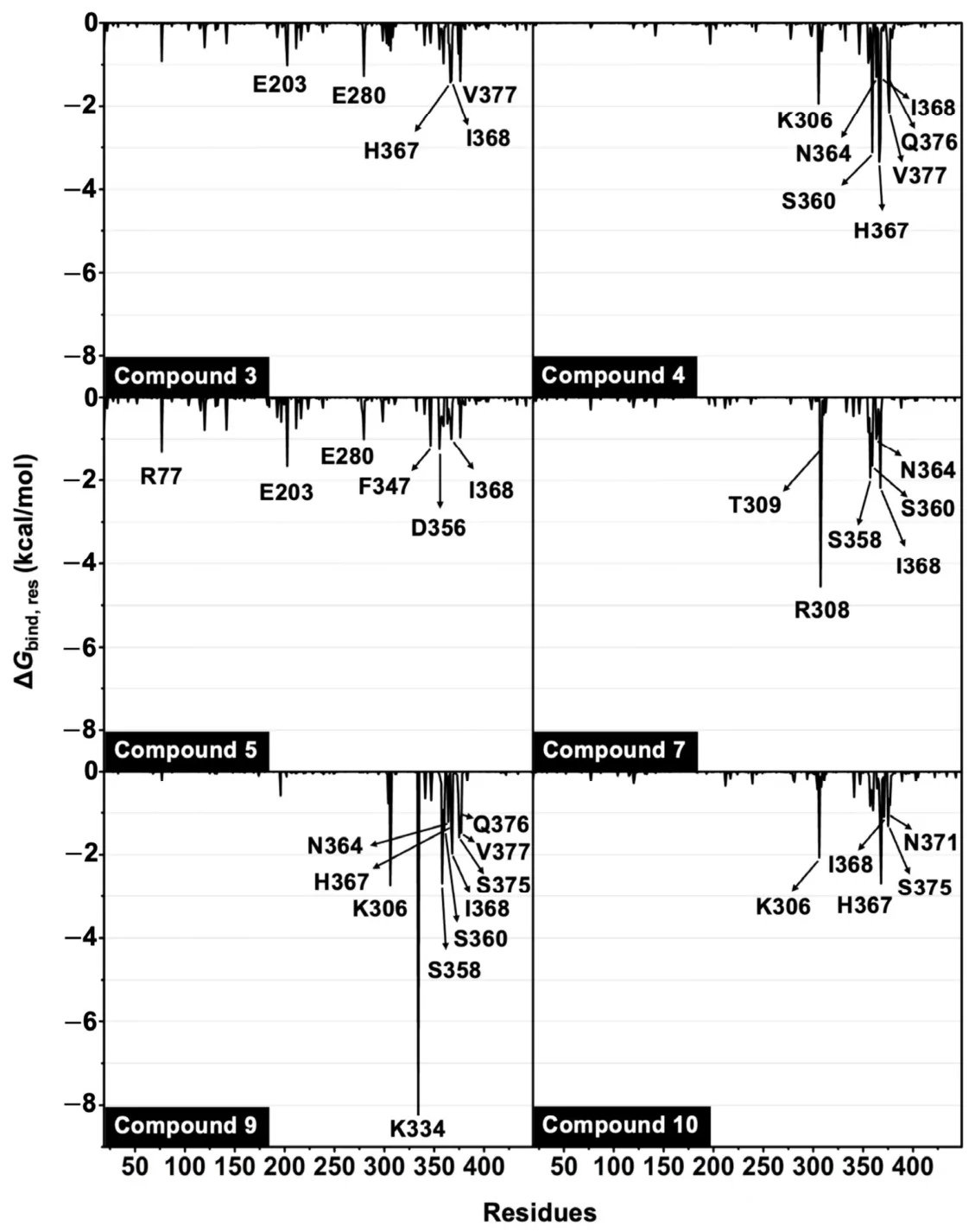
MM/PBSA-based Δ*G*_bind,res_ of six aminononaphthoquinone–chalcone hybrids in complex with human tyrosinase.

**Figure 7:**
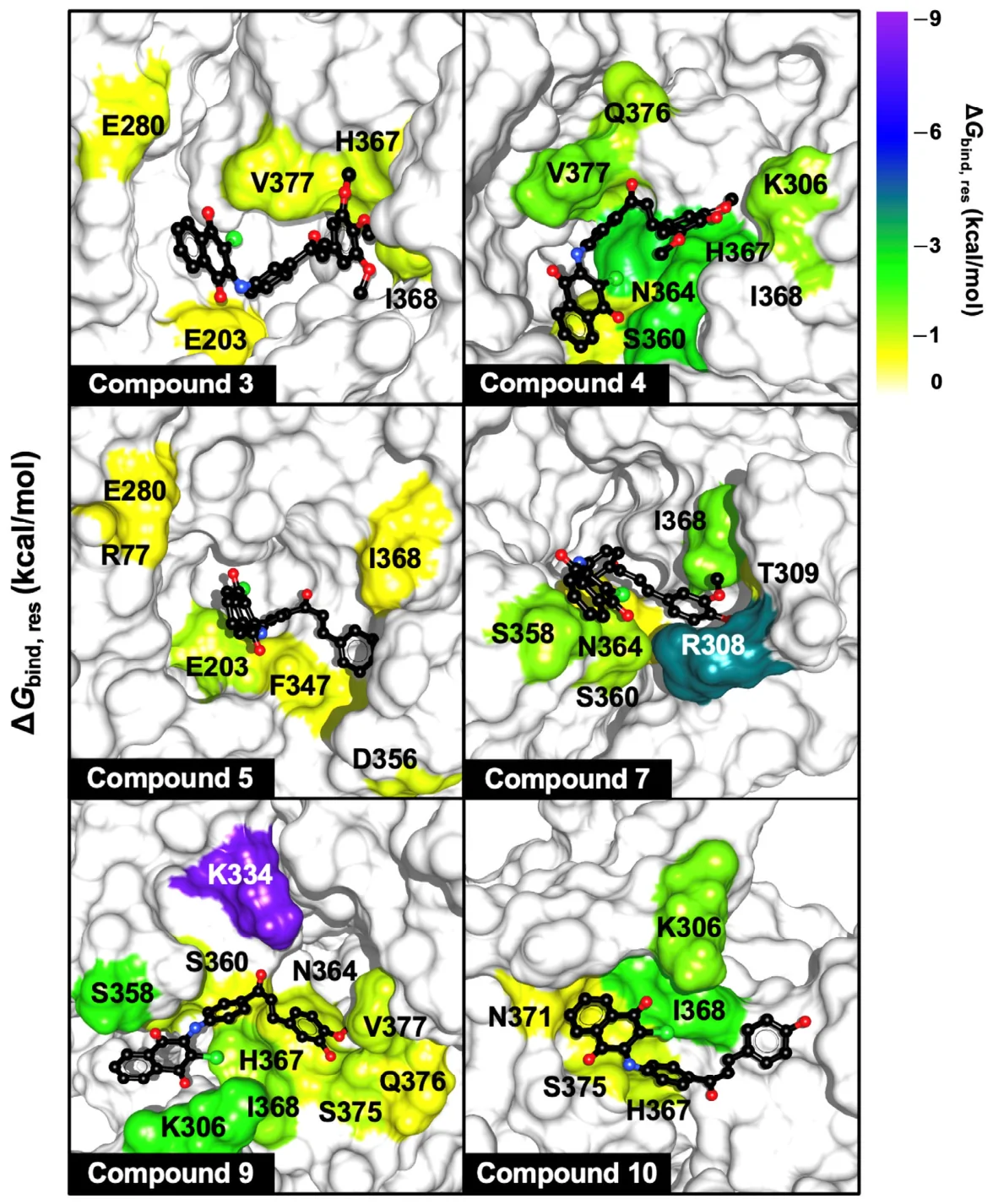
Binding pattern of six aminononaphthoquinone–chalcone hybrids within the active site of human tyrosinase captured from the final MD snapshot. The catalytic coppers are hidden. The key binding residues are colored according to their Δ*G*_bind,res_ values with the color gradient ranging from white (highest Δ*G*_bind,res_) to purple (lowest Δ*G*_bind,res_).

Three independent MD replicas of the human tyrosinase complexes with compounds **4**, **7**, and **9** were performed to validate the reproducibility of the ligand–protein interactions. The #Contacts and #H-bonds analyses revealed highly consistent interaction patterns across the independent trajectories (Figure S11). Among the three compounds, compound **9** exhibited the most favorable interaction profile, characterized by the highest numbers of protein–ligand contacts and H-bonds. These findings are further supported by the key binding residue analyses (Figure 6 and Figure 7) and Δ*G*_bind_ calculations (Table 2), as discussed in the following sections.

### 2.4. Key Binding Residues

To identify the amino acid residues involved in the binding of the six aminonaphthoquinone–chalcone hybrids to human tyrosinase, per-residue binding free energy decomposition (Δ*G*_bind,res_) was performed using the MM/PBSA method on 100 snapshots extracted from the final 10 ns of the MD simulations. The results are presented in Figure 6. Key binding residues with Δ*G*_bind,res_ values of ≤ −1.0 kcal/mol are shown in Figure 7.

The analysis revealed that all six compounds interacted with residues located within the active site of human tyrosinase. Compound **4** formed favorable interactions with seven residues (K306, S360, N364, H367, I368, Q376, and V377), whereas compound **5** interacted with six residues (R77, E203, E208, F347, D356, and I368), and compound **7** also engaged six residues (R308, T309, S358, S360, N364, and I368). Notably, the compound **9**–human tyrosinase complex exhibited the largest network of stabilizing interactions, involving 10 key residues (K306, K334, S358, S360, N364, H367, I368, S375, Q376, and V377). Among these, K334 made the strongest energetic contribution, with a Δ*G*_bind, res_ value of −8.25 kcal/mol (Figure 6). In addition, compounds **3**, **4**, **9**, and **10** interacted with the catalytic residue H367. Among all investigated compounds, compound **9** displayed the greatest number of hotspot residues, consistent with its most favorable Δ*G*_bind_ (Table 2).

Overall, these findings demonstrate that all six aminonaphthoquinone–chalcone hybrids are capable of binding within the active site of human tyrosinase. The observed binding mode is consistent with those reported for known human tyrosinase inhibitors, including Thiamidol™ [28], amphotericin B [29], epicatechin gallate, epigallocatechin gallate [35], kojic acid, arbutin [16], coumarin derivative C9, and vanillin derivative V7 [16].

### 2.5. Predicted Binding Free Energy

To evaluate the binding affinity of the six aminonaphthoquinone–chalcone hybrids toward human tyrosinase, the Δ*G*_bind_ and its energy components were calculated using the MM/PBSA method [36] based on 100 snapshots extracted from the final 10 ns of the MD simulations. As summarized in Table 2, the molecular mechanics energy (Δ*E*_MM_) in the gas phase revealed that vdW interactions (Δ*E*_vdW_) were the primary driving force for ligand binding, whereas electrostatic interactions (Δ*E*_ele_) made a comparatively smaller contribution. This finding agreed well with the previous studies showing that vdW interactions dominate the binding of Thiamidol™, catechin, epicatechin gallate, and epigallocatechin gallate to the catalytic site of human tyrosinase [28,35].

The calculated Δ*G*_bind_ values of the six aminonaphthoquinone–chalcone hybrids ranged from −20.32 to −38.38 kcal/mol (Table 2). Among the investigated compounds, compound **9** exhibited the most favorable Δ*G*_bind_ (−38.38 ± 0.67 kcal/mol), followed by compound **4** (−35.00 ± 0.62 kcal/mol), compound **7** (−34.40 ± 0.34 kcal/mol), compound **10** (−29.99 ± 0.39 kcal/mol), compound **3** (−29.55 ± 0.32 kcal/mol), and compound **5** (−20.32 ± 0.42 kcal/mol).

To further validate the reliability of the Δ*G*_bind_ estimates, MM/PBSA calculations were performed for the human tyrosinase complexes with compounds **4**, **7**, and **9** using three independent MD simulations. As summarized in Table S2, all three compounds consistently exhibited favorable Δ*G*_bind_ values across independent simulations, with average Δ*G*_bind_ values of −33.59, −32.80, and −38.56 kcal/mol for compounds **4**, **7**, and **9**, respectively. Notably, compound **9** demonstrated a higher binding affinity than the other compounds, which agrees well with the structural stability (Figure 4A), compactness (Figure 4B), #Contacts (Figure 5A), #H-bonds (Figure 5B), and key binding residue (Figure 6 and Figure 7) results as mentioned above.

### 2.6. Drug Likeness

Prediction of drug-likeness is an important step in early-stage drug discovery, as it helps evaluate the likelihood that candidate compounds possess favorable physicochemical properties for further development [37]. In this study, the drug-likeness of the six selected aminonaphthoquinone–chalcone hybrids was evaluated using the SwissADME online platform [38]. Key molecular descriptors, including molecular weight (MW), the number of hydrogen bond donors and acceptors (HBD and HBA, respectively), the number of rotatable bonds (RBs), topological polar surface area (TPSA), and lipophilicity (log *P*), were assessed. As summarized in Table 3, all six compounds satisfied Lipinski’s rule of five, including (i) MW < 500 Da, (ii) HBD ≤ 5 and HBA ≤ 10, (iii) RB ≤ 10, (iv) TPSA ≤ 140 Å², and (v) log *P* ≤ 5 [39,40]. These results suggest that the six aminonaphthoquinone–chalcone hybrids possess favorable drug-like properties and therefore represent promising candidates for further development as human tyrosinase inhibitors.

## 3. Materials and Methods

### 3.1. System Preparation and Molecular Docking

The FASTA sequence of human tyrosinase was obtained from the National Center for Biotechnology Information (GenBank: AAB60319.1). A homology model of human tyrosinase was constructed using the SWISS-MODEL server with the crystal structure of human tyrosinase-related protein 1 in complex with kojic acid (PDB ID: 5M8Q) [4] as the template. Following model construction, the two Zn^2+^ ions in the template were replaced with Cu^2+^ ions (CuA and CuB), while preserving the experimentally resolved coordination geometry with the six conserved catalytic histidine residues (His180, His202, and His211 coordinating CuA, and His363, His367, and His390 coordinating CuB).

The protonation state of human tyrosinase at physiological pH (7.4) was predicted using the PDB2PQR web server [41]. The three-dimensional (3D) structures of the aminonaphthoquinone–chalcone hybrids were generated according to the previous report [26] using GaussView 6 (Semichem Inc., Shawnee Mission, KS, USA) [42]. Each ligand was subsequently subjected to full geometry optimization at the 6-31G(d) level of theory using Gaussian 09 [43] to obtain its lowest-energy conformation. The protonation state of each ligand was determined at pH 7.4 using Marvinsketch version 22.6.5 (ChemAxon Ltd., Budapest, Hungary). Following established procedures [44,45,46,47,48], electrostatic potential (ESP) charges were then calculated for the optimized ligand structures at the HF/6-31G(d) level of theory using Gaussian 09 [43]. Molecular docking was performed using the CB-Dock2 web server [49], and the resulting binding poses were analyzed with BIOVIA Discovery Studio Visualizer 2024 (v24.1.0.23298; Dassault Systèmes BIOVIA Corp., San Diego, CA, USA) [50].

### 3.2. MD Simulation

All-atom MD simulation of each protein–ligand complex was performed using the AMBER20 software package (University of California, San Francisco, CA, USA [51]. The bonded and non-bonded parameters for all ligands were assigned using the General Amber Force Field (GAFF) [52], whereas the protein was parameterized with the AMBER ff14SB force field [53]. Missing hydrogen atoms were added using the LEaP module. The catalytic copper ions were treated as non-bonded Cu^2+^ ions using the copper ion parameters available in the AMBER LEaP ion library. Each system was solvated in a periodic TIP3P water box [54] and Na^+^ ions were added to neutralize the system. Simulations were carried out under isothermal–isobaric (NPT) conditions at 310 K and 1 atm. The SHAKE algorithm [55] was applied to constrain all bonds involving hydrogen atoms. A residue-based cutoff of 12 Å was used for non-bonded interactions, and long-range electrostatic interactions were treated using the particle mesh Ewald (PME) method [56]. 

Each system was first subjected to energy minimization using the steepest descent (SD) and conjugate gradient (CG) methods for 1,000 and 2,000 steps, respectively, to remove unfavorable contacts. The systems were then gradually heated to 310 K over 100 ps, followed by 5 ns of restrained MD simulations with progressively reduced positional restraints (100, 50, 20, 10, 5, and 1 kcal/mol·Å²). After that the unrestrained MD simulations were conducted in the NPT ensemble (1 atm and 310 K) until reaching 70 ns. Snapshots collected during the final 10 ns (60–70 ns) of each trajectory were used for subsequent analyses.

### 3.3. Structural Analysis and Binding Free Energy Calculation of Ligand–Protein Complexes

The CPPTRAJ [57] in the AMBER20 package was used to analyze the structural and dynamic properties of the protein–ligand complexes, including RMSD, Rg, #Contacts, and H-bond formation. Moreover, the MM/PBSA calculations [36] were employed to estimate the binding affinity and identify key stabilizing amino acid residues involved in the ligand binding. The MM/PBSA calculations were performed using an interior dielectric constant of 5.0 without normal-mode analysis, based on 100 snapshots extracted from the final 10 ns of the MD trajectories.

### 3.4. Drug Likeness Prediction

The drug-likeness of aminonaphthoquinone–chalcone hybrids was evaluated using the SwissADME web server (www.swissadme.ch/) (accessed on 11 October 2025) [38].

## 4. Conclusions

In this study, we investigated the binding characteristics of 10 aminonaphthoquinone–chalcone hybrids toward human tyrosinase using molecular docking, MD simulations, and MM/PBSA Δ*G*_bind_ calculations. Molecular docking identified compounds **3**, **4**, **5**, **7**, **9**, and **10** as the most promising candidates, exhibiting higher predicted binding affinity than the remaining hybrids. Subsequent MD simulations demonstrated that, among the investigated complexes, the compound **9**–human tyrosinase complex exhibited the highest numbers of protein–ligand contacts and H-bonds, suggesting the most stable interaction profile. MM/PBSA calculations further revealed that vdW interactions were the dominant driving force for ligand binding. Notably, compound **9** displayed the most favorable Δ*G*_bind_ (−38.38 ± 0.67 kcal/mol) and interacted with the greatest number of active-site residues (K306, K334, S358, S360, N364, H367, I368, S375, Q376, and V377). The reproducibility of these findings was confirmed by three independent MD simulations, which consistently supported the structural stability and favorable binding of compounds **4**, **7**, and particularly **9**. In addition, all six selected compounds satisfied Lipinski’s rule of five, indicating favorable drug-like properties. Collectively, these findings identify compound **9** as the most promising aminonaphthoquinone–chalcone hybrid for further experimental evaluation as a potential human tyrosinase inhibitor. Nevertheless, experimental studies are required to validate its inhibitory activity and therapeutic potential.

## Figures and Tables

**Table 1:** CB-Dock2 binding energies of complexes between the 10 aminonaphthoquinone–chalcone hybrids and human tyrosinase.

Compound	CB-Dock2 Binding Energy (kcal/mol)
**1**	−8.1
**2**	−8.1
**3**	−8.4
**4**	−9.2
**5**	−8.4
**6**	−8.3
**7**	−8.4
**8**	−7.5
**9**	−8.4
**10**	−8.5

**Table 2:** Δ*G*_bind_ and their energy components calculated using the MM/PBSA method for six aminonaphthoquinone–chalcone hybrids in complex with human tyrosinase. Data are shown as the mean ± standard error of the mean (SEM) from 100 snapshots extracted from the last 10 ns of the simulation.

Energy Component	Compound 3	Compound 4	Compound 5	Compound 7	Compound 9	Compound 10
**Δ*E*_vdW_**	−39.11 ± 0.37	−36.10 ± 0.62	−33.58 ± 0.48	−35.92 ± 0.35	−36.57 ± 0.69	−32.12 ± 0.44
**Δ*E*_ele_**	0.99 ± 0.15	−4.74 ± 0.22	1.38 ± 0.20	0.21 ± 0.15	−4.19 ± 0.18	1.26 ± 0.20
**Δ*E*_MM_**	−38.12 ± 0.43	−40.84 ± 0.67	−32.20 ± 0.48	−35.70 ± 0.38	−40.76 ± 0.65	−30.86 ± 0.44
**Δ*G*_solv,nonpolar_**	−6.51 ± 0.05	−5.94 ± 0.05	−5.55 ± 0.07	−5.61 ± 0.04	−6.48 ± 0.06	−5.56 ± 0.05
**Δ*G*_solv,polar_**	15.07 ± 0.30	11.78 ± 0.24	17.43 ± 0.36	6.92 ± 0.19	8.86 ± 0.13	6.43 ± 0.24
**Δ*G*_solv_**	8.57 ± 0.27	5.85 ± 0.22	11.88 ± 0.33	1.31 ± 0.15	2.38 ± 0.14	0.87 ± 0.22
**Δ*G*_bind_**	−29.55 ± 0.32	−35.00 ± 0.62	−20.32 ± 0.42	−34.40 ± 0.34	−38.38 ± 0.67	−29.99 ± 0.39

**Table 3:** Predicted drug-likeness of compounds **3**, **4**, **5**, **7**, **9**, and **10** according to Lipinski’s rule of five.

Compound	Lipinski’s Rule of Five						
	MW (≤500 Da)	HBD (≤5)	HBA (≤10)	RB (≤10)	TPSA (≤140 Å^2^)	Log *P* (≤5)	Drug–Likeness
**3**	503.93	1	6	8	90.93	1.65	Yes
**4**	489.90	2	6	7	101.93	1.46	Yes
**5**	429.85	2	4	5	83.47	2.13	Yes
**7**	473.90	1	5	7	81.70	1.99	Yes
**9**	459.88	2	5	6	92.70	1.79	Yes
**10**	429.85	2	4	5	83.47	2.13	Yes

MW, molecular weight; HBD, number of hydrogen bond donors; HBA, number of hydrogen bond acceptors; RB, number of rotatable bonds; TPSA, topological polar surface area; log *P*, lipophilicity.

## Data Availability

The data presented in this study are available within the article and Supplementary Materials.

## Supplementary Materials

The following supporting information can be downloaded at: https://www.mdpi.com/article/10.3390/ijms27167328/s1.
