# Supplementary material for: Structural Dynamics and Susceptibility of Aminonaphthoquinone–Chalcone Hybrids Against Human Tyrosinase
Source: Int J Mol Sci. 2026 Aug 17;27(16):7328. doi: 10.3390/ijms27167328 (PMC13513844; doi:10.3390/ijms27167328)
Supplement: Supplementary file 1 [file ijms-27-07328-s001.zip › ijms-4277596-supplementary.pdf]

Supporting Data

Manuscript Title: Structural Dynamics and Susceptibility of Aminonaphthoquinone–Chalcone Hybrids Against Human Tyrosinase

Sahachai Sabuakham[1⊥], Napat Nuramrum[1⊥], Thanyada Rungrotmongkol[2, 3], Ratchanok Pingaew[4], Panupong Mahalapbutr[1, *]

[1]Department of Biochemistry, Center for Translational Medicine, Faculty of Medicine, Khon Kaen University, Khon Kaen 40002, Thailand

[2]Program in Bioinformatics and Computational Biology, College of Interdisciplinary and Integrative Studies, Chulalongkorn University, Bangkok 10330, Thailand

[3]Center of Excellence in Structural and Computational Biology, Department of Biochemistry, Faculty of Science, Chulalongkorn University, Bangkok 10330, Thailand

[4]Department of Chemistry, Faculty of Science, Srinakharinwirot University, Bangkok 10110, Thailand

⊥These authors contributed equally to this work.

*Corresponding author. E-mail: panupma@kku.ac.th (P. Mahalapbutr)

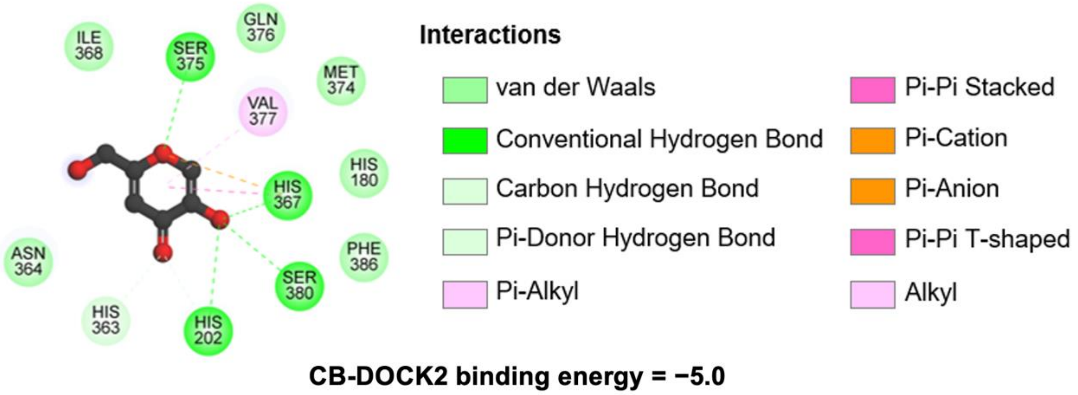

Figure S1. 2D interaction profile of kojic acid within the active site of human tyrosinase.

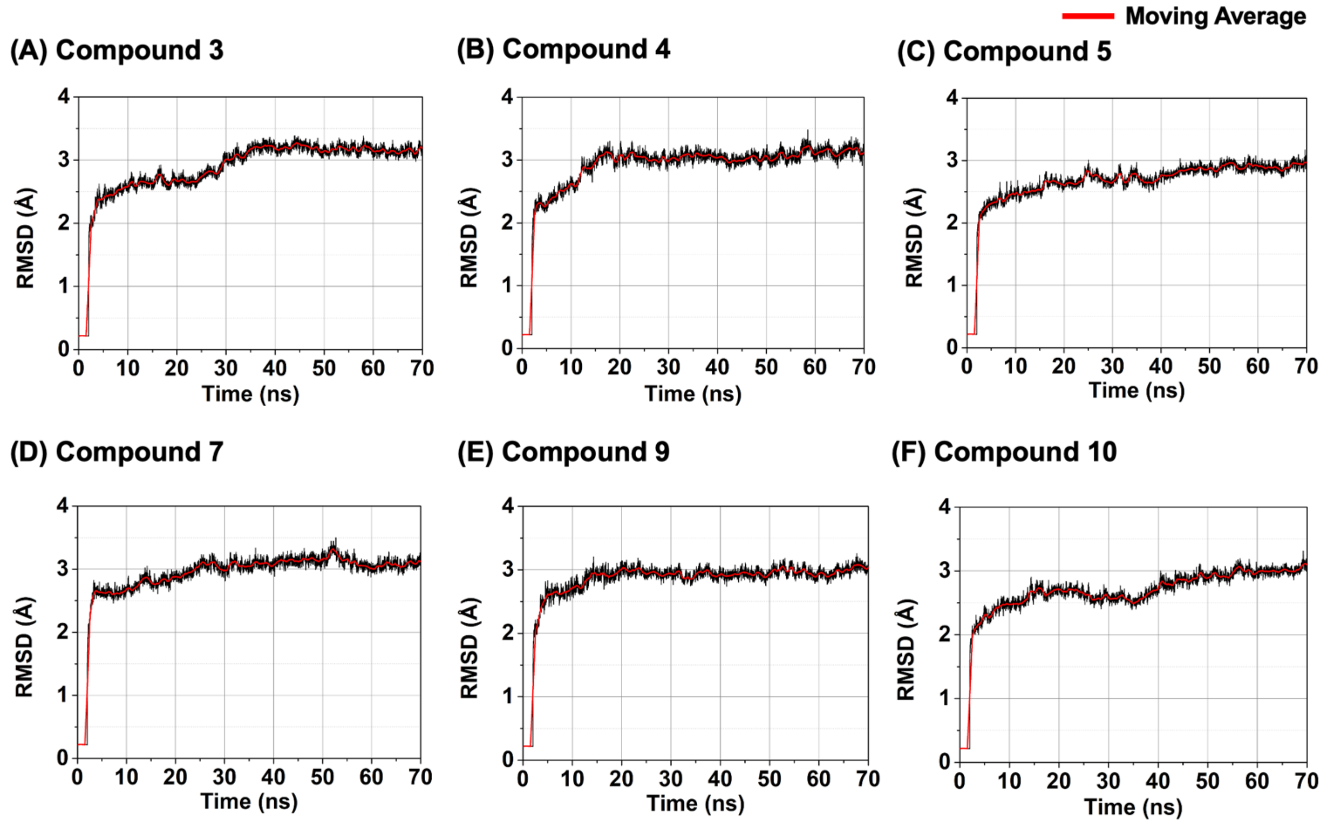

Figure S2. Time evolution of RMSD profiles for six aminonaphthoquinone–chalcone hybrids in complex with human tyrosinase obtained from MD simulations. Black lines represent raw RMSD values, while red lines indicate moving average RMSD profiles.

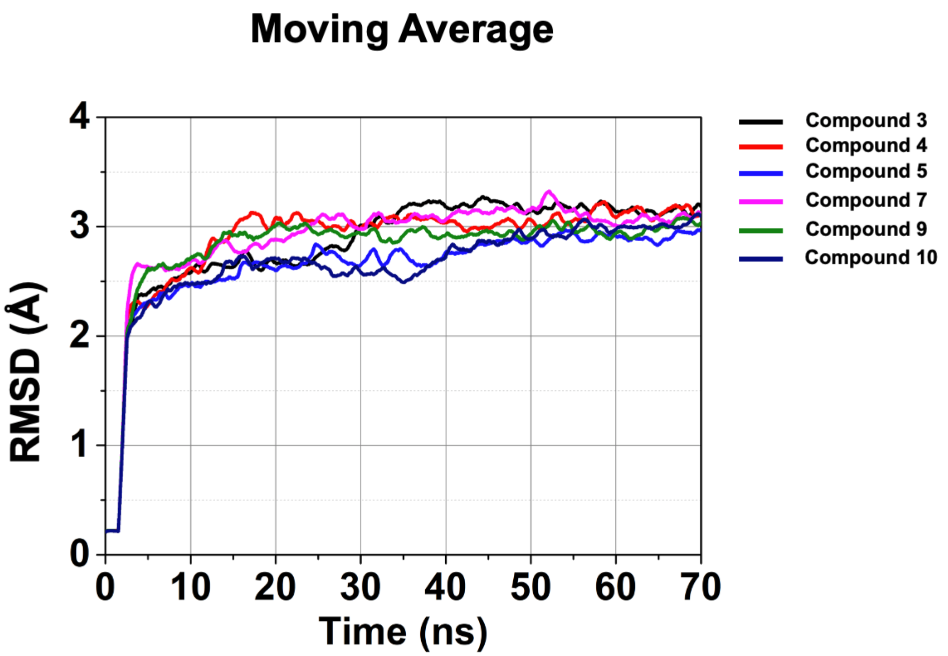

Figure S3. Comparative moving average RMSD profiles for six aminonaphthoquinone–chalcone hybrids in complex with human tyrosinase obtained from MD simulations.

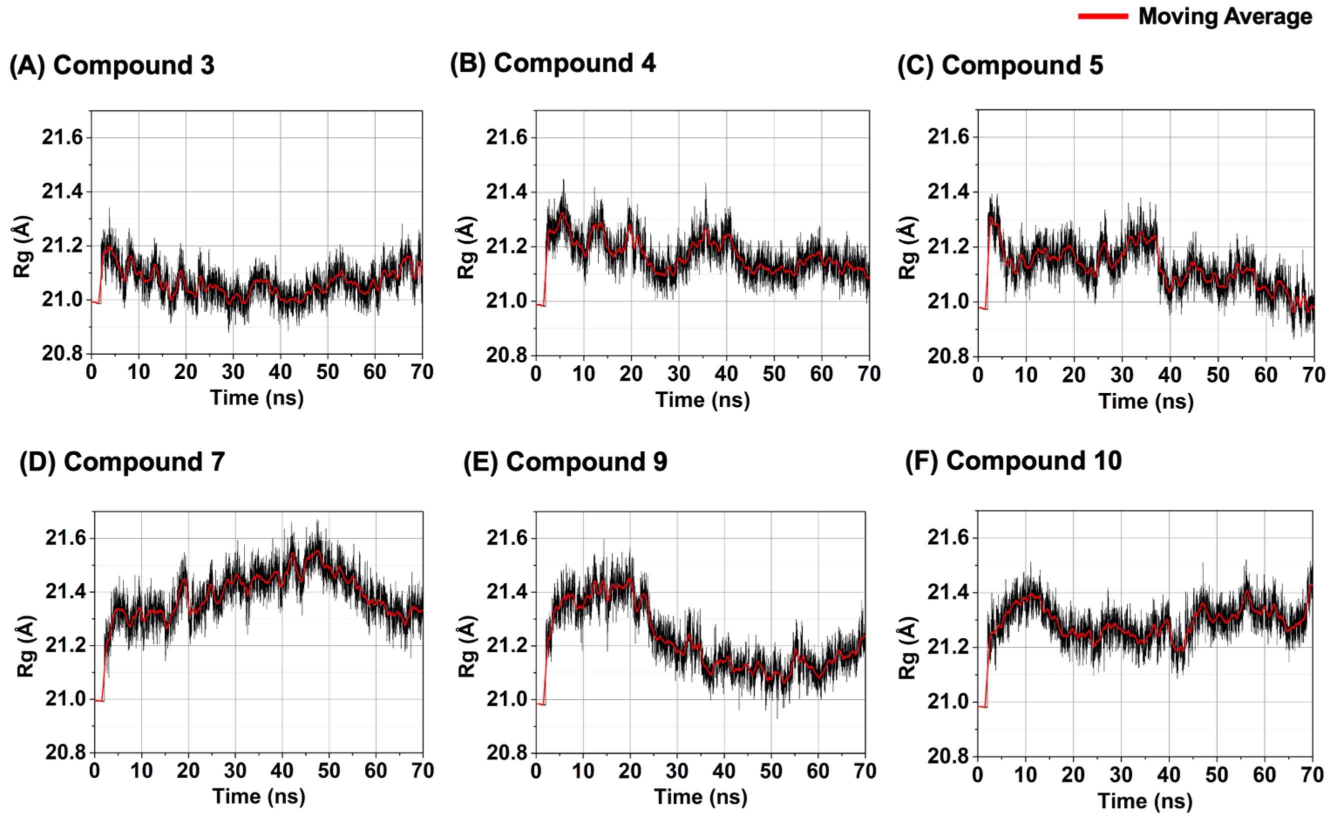

Figure S4. Time evolution of Rg profiles for six aminonaphthoquinone–chalcone hybrids in complex with human tyrosinase obtained from MD simulations. Black lines represent raw Rg values, while red lines indicate moving average Rg profiles.

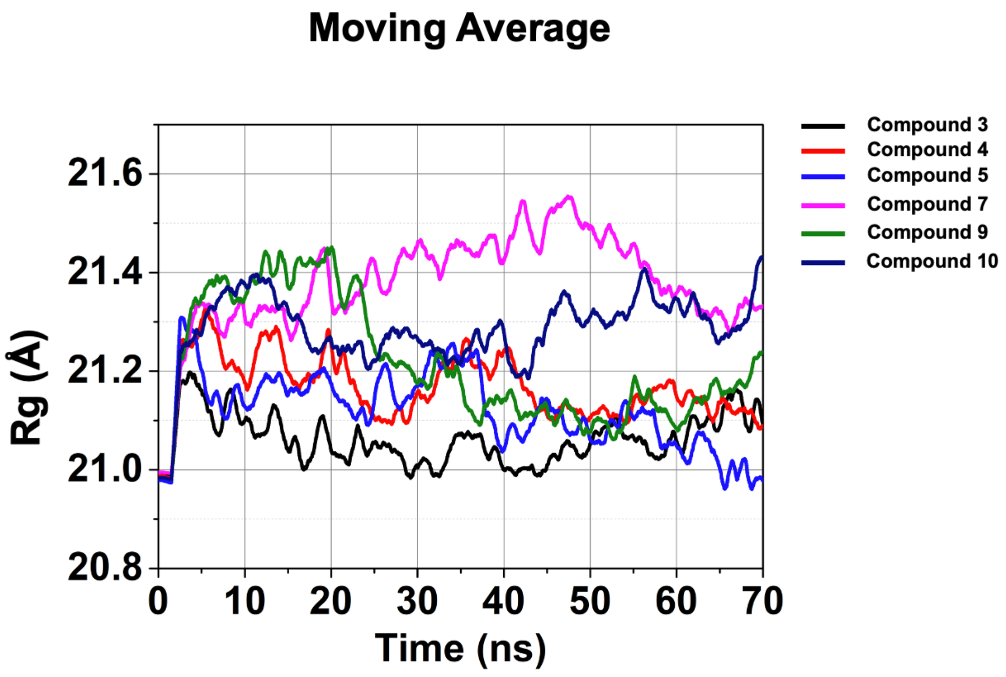

Figure S5. Comparative moving average Rg profiles for six aminonaphthoquinone–chalcone hybrids in complex with human tyrosinase obtained from MD simulations.

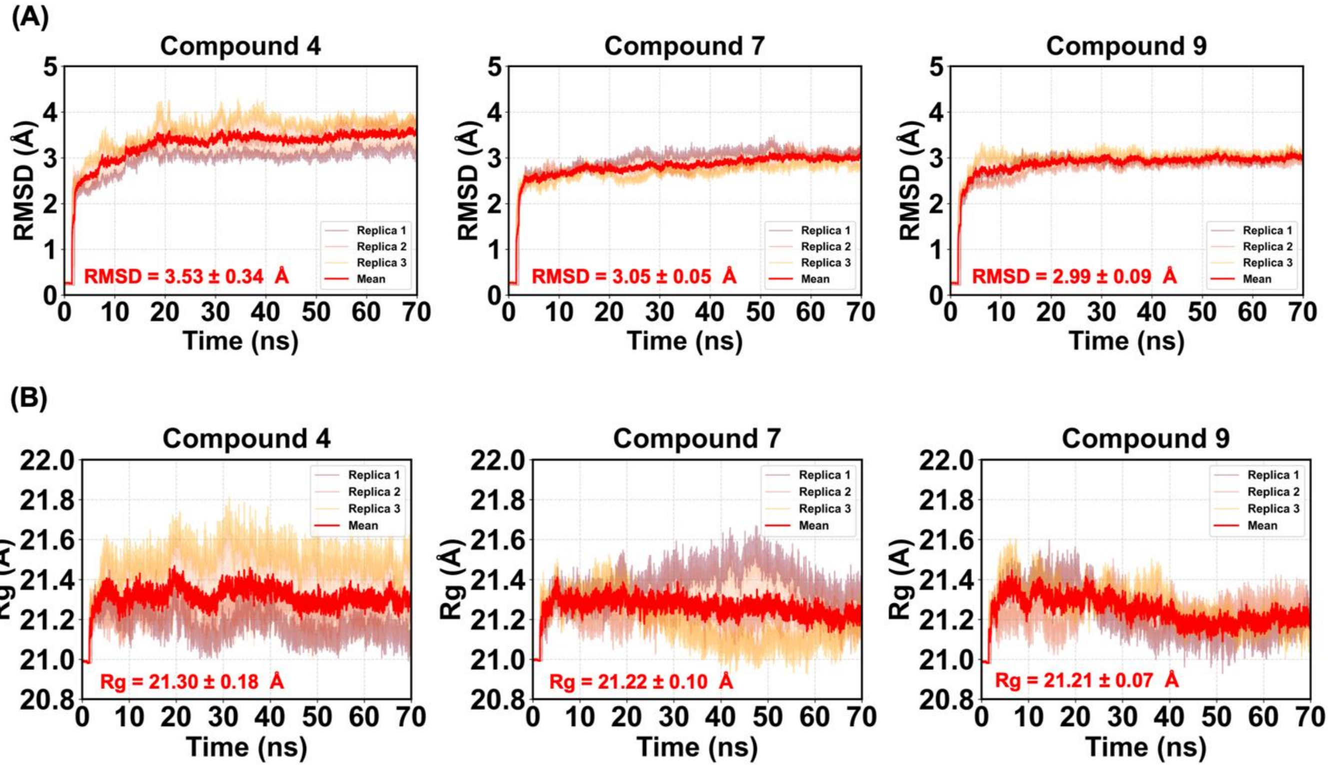

Figure S6. Time evolution of **(A)** RMSD and **(B)** Rg for compounds **4, 7,** and **9** in complex with human tyrosinase obtained from 70 ns MD simulations (three replicas). The average values were calculated from the last 10 ns of replicas 1-3.

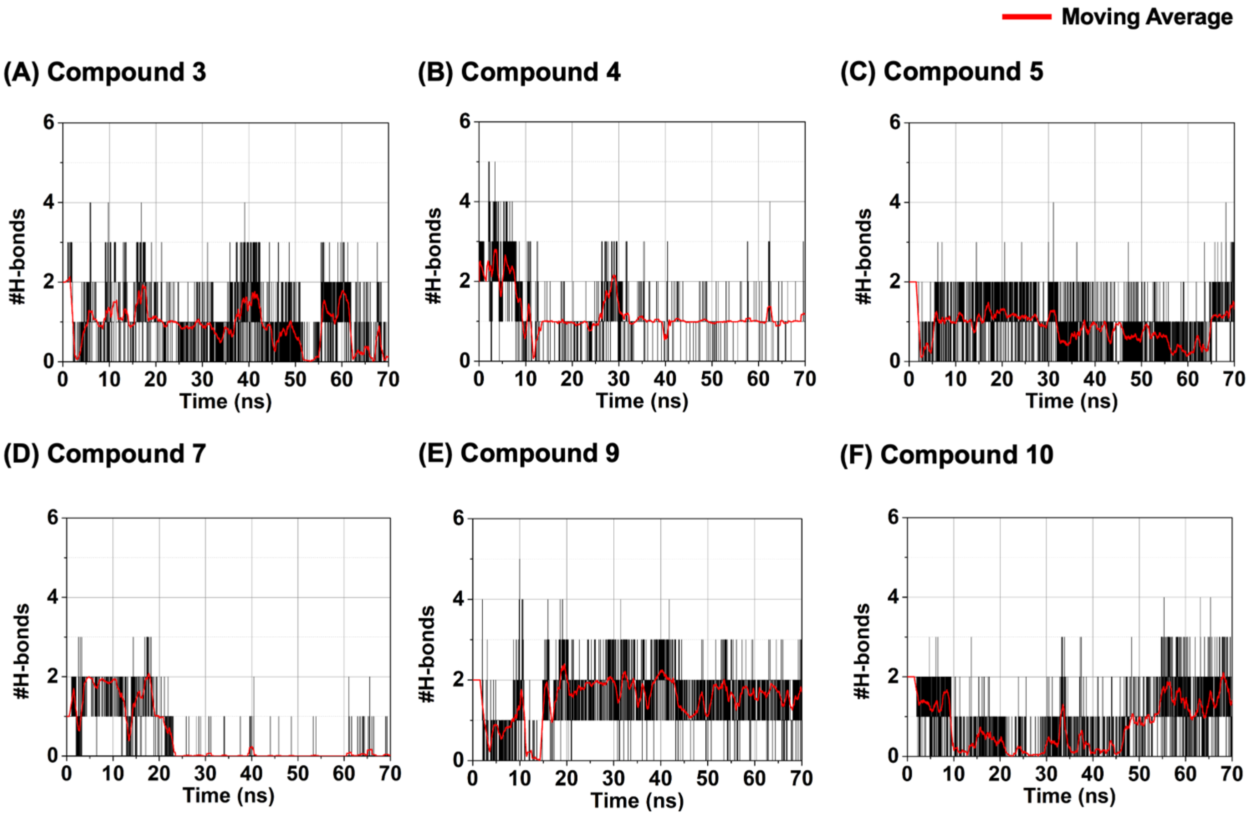

Figure S7. Time evolution of #H-bonds profiles for six aminonaphthoquinone–chalcone hybrids in complex with human tyrosinase obtained from MD simulations. Black lines represent raw #H-bonds values, while red lines indicate moving average #H-bonds profiles.

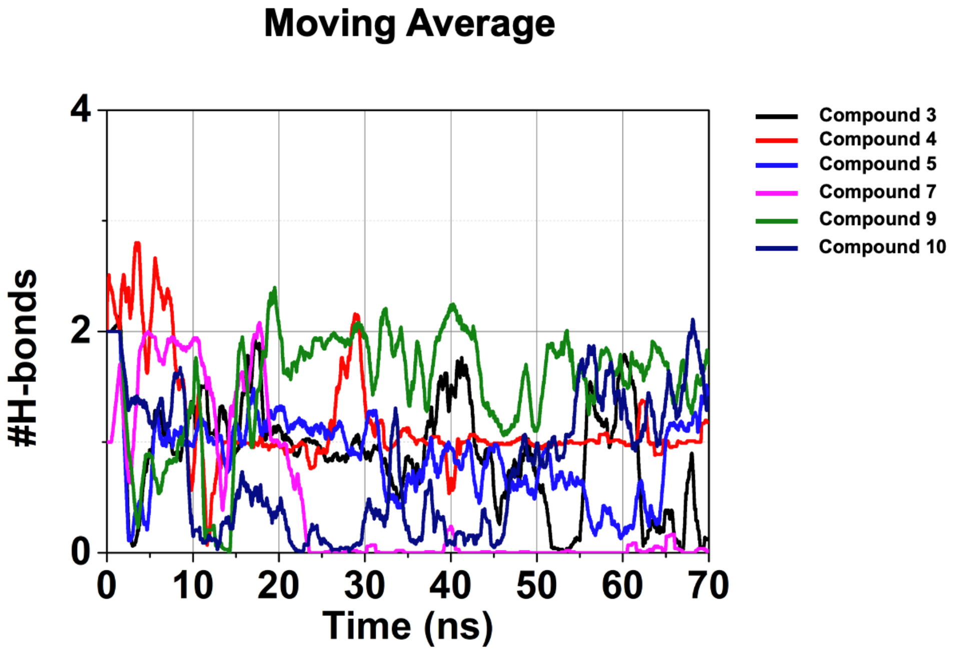

Figure S8. Comparative moving average #H-bonds profiles for six aminonaphthoquinone–chalcone hybrids in complex with human tyrosinase obtained from MD simulations.

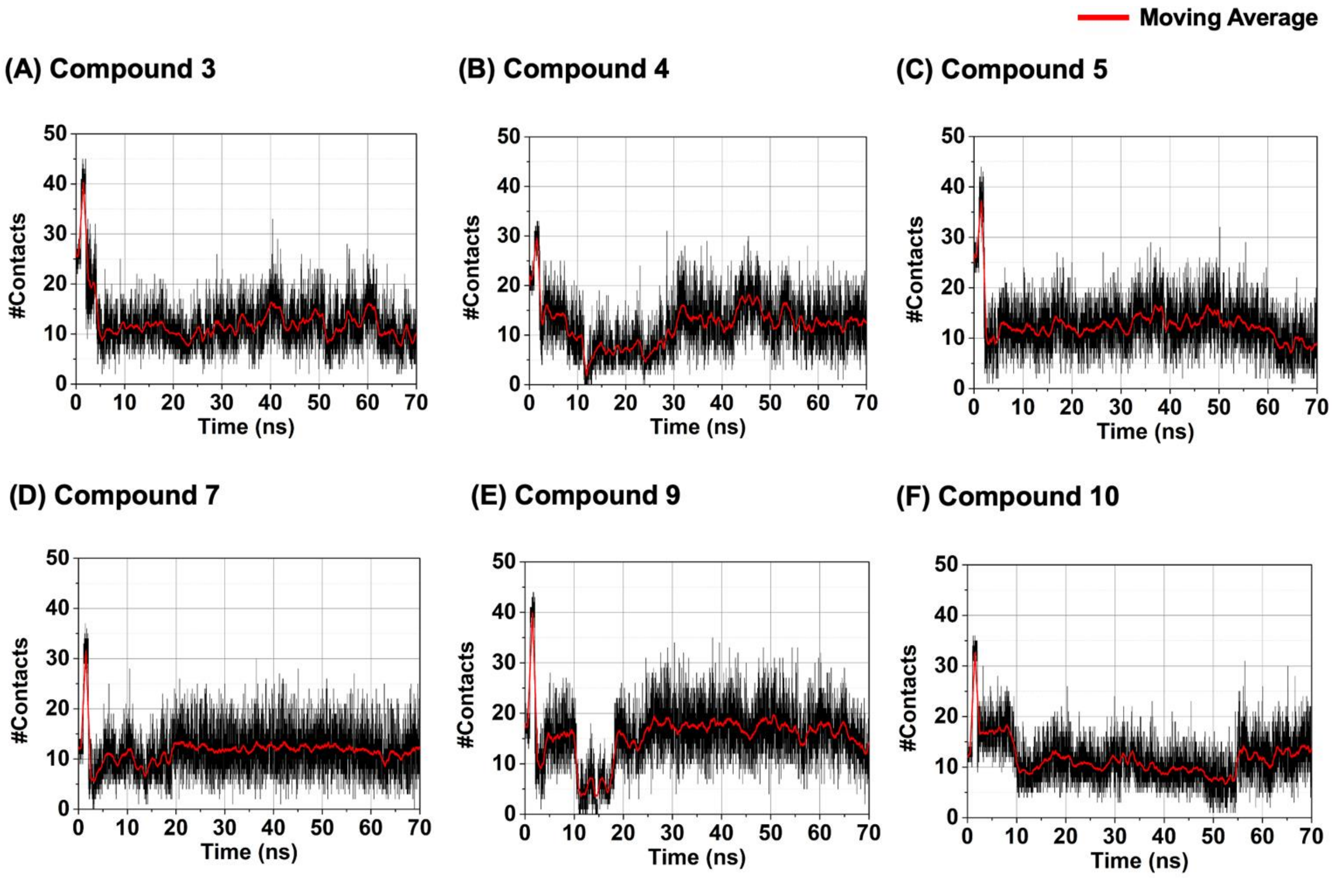

Figure S9. Time evolution of #Contacts profiles for six aminonaphthoquinone–chalcone hybrids in complex with human tyrosinase obtained from MD simulations. Black lines represent raw #Contacts values, while red lines indicate moving average #Contacts profiles.

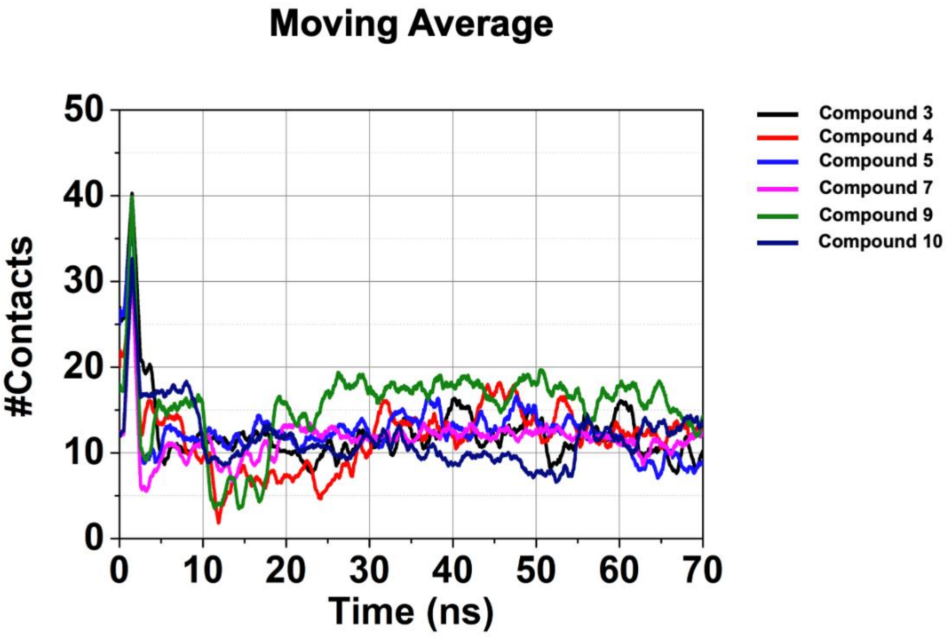

Figure S10. Comparative moving average #Contacts profiles for six aminonaphthoquinone–chalcone hybrids in complex with human tyrosinase obtained from MD simulations.

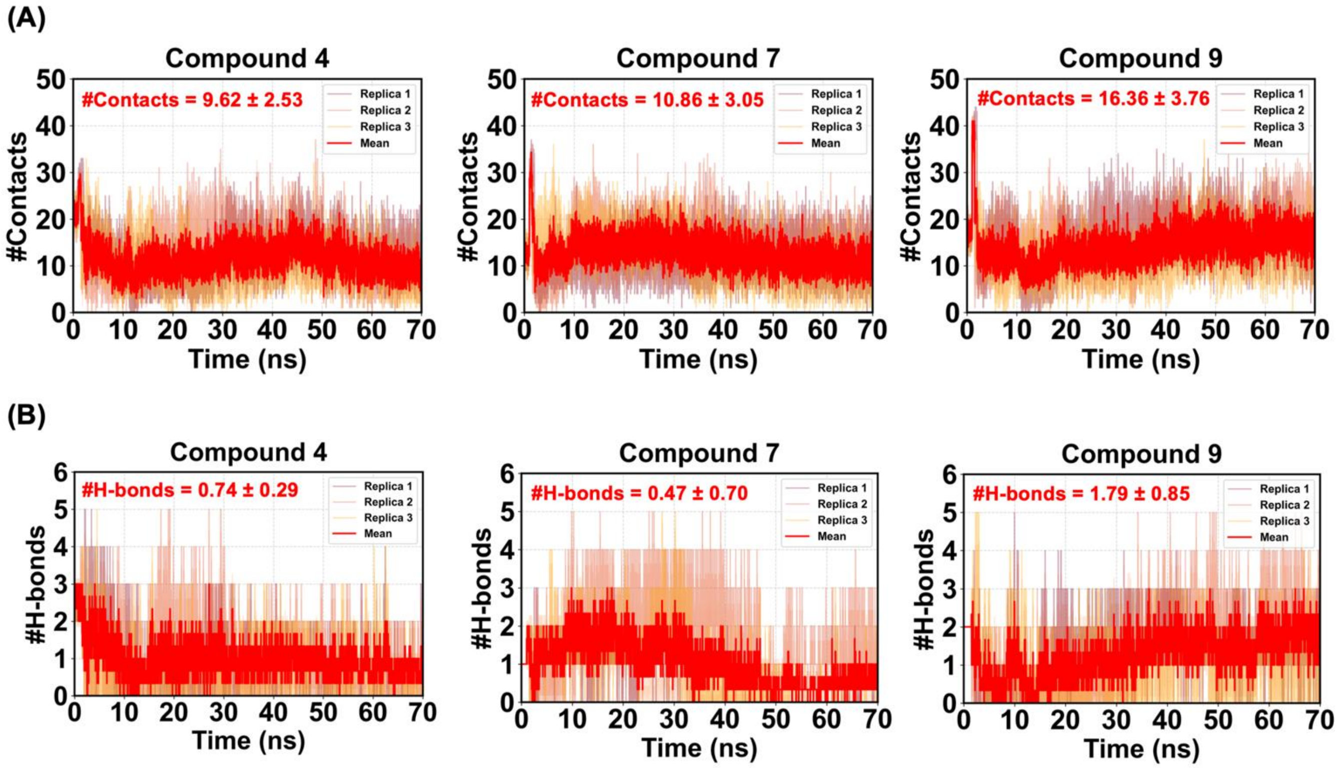

Figure S11. Time evolution of **(A)** #Contacts and **(B)** #H-bonds for compounds **4, 7,** and **9** in complex with human tyrosinase obtained from 70 ns MD simulations (three replicas). The average values were calculated from the last 10 ns of replicas 1-3.

Table S1. Hydrogen bond occupancy analysis of six aminonaphthoquinone–chalcone hybrids in complex with human tyrosinase.

Acceptor	Donor–H	Donor	Frames	Occupancy (%)	Avg Dist (Å)	Avg Angle (°)
**Compound 3**						
MOL@O3	SER_307@HG	SER_307@OG	568	28.40	3.01	144.26
MOL@O5	SER_307@HG	SER_307@OG	444	22.20	3.11	145.99
**Compound 4**						
MOL@O2	SER_360@HG	SER_360@OG	1957	97.85	2.83	155.05
**Compound 5**						
ALA_357@O	MOL@H14	MOL@O4	634	31.70	2.84	157.17
MOL@O2	GLN_378@HE21	GLN_378@NE2	247	12.35	3.02	144.92
**Compound 7**						
N/A						
**Compound 9**						
SER_375@O	MOL@H18	MOL@O2	1734	86.70	2.88	148.84
MOL@O1	LYS_334@HZ1	LYS_334@NZ	478	23.90	2.88	148.54
MOL@O1	LYS_334@HZ2	LYS_334@NZ	462	23.10	2.90	148.87
MOL@O1	LYS_334@HZ3	LYS_334@NZ	460	23.00	2.88	149.79
**Compound 10**						
SER_375@O	MOL@H9	MOL@N1	1257	62.85	3.12	140.61
ALA_357@O	MOL@H15	MOL@O4	1093	54.65	2.83	154.66

Data are derived from the last 10 ns of the MD simulation. Occupancy (%) represents the percentage of simulation frames in which each hydrogen bond was observed. Rows highlighted in yellow indicate H-bonds with occupancy ≥ 10%.

MOL = ligand molecule; Avg Dist = average H-bond donor–acceptor distance (Å); Avg Angle = average H-bond angle (°). H-bond criteria: distance ≤ 3.5 Å and angle ≥ 120°. A total of 2,000 simulation frames from the last 10 ns were analyzed.

Table S2. MM/PBSA-based binding free energy (ΔG_{bind}) of three selected aminonaphthoquinone–chalcone hybrids in complex with human tyrosinase from three independent MD simulations.

Compound	ΔG_{bind} (kcal/mol)			Avg ΔG_{bind} (kcal/mol)	SD
	Replica 1	Replica 2	Replica 3		
**Compound 4**	−35.00 ± 0.62	−30.82 ± 0.29	−34.94 ± 0.38	**−33.59**	2.40
**Compound 7**	−34.40 ± 0.34	−32.18 ± 0.27	−31.81 ± 0.45	**−32.80**	1.40
**Compound 9**	−38.38 ± 0.67	−39.73 ± 0.39	−37.57 ± 0.41	**−38.56**	1.09
